# Asthma diagnosis and treatment – 1001. Identification of prevalent sensitizing allergens in India

**DOI:** 10.1186/1939-4551-6-S1-P1

**Published:** 2013-04-23

**Authors:** Shubnum Singh

**Affiliations:** 1 Allied Health & Wellness Programs, New Delhi, India

## Background

This study was conducted to identify the most common sensitizing food and inhalant allergens in physician-diagnosed allergic children and adults in North India.

## Methods

274 allergy-diagnosed patients, divided into Group A (aged 6–12 yrs) and Group B (aged 12–65 yrs), were enrolled in the study. They were classified as atopic if had at least one positivity when screened with ImmunoCAP^®^ Phadiatop and fx5 (Food mix 5; 6 common foods), a technology considered as the gold standard for IgE antibody blood testing worldwide. For identification of the sensitizing allergens atopic patients were further tested by ImmunoCAP^®^ Specific IgE using a broad panel of common Indian allergens covering 17 foods and 19 inhalants (singles/mixes). Total IgE level was also determined for each atopic patient.

## Results

Phadiatop/fx5 determined 59% (162/274) of the patients as atopic, where of 159 were included in further evaluation; 10% were in Group A and 90% were in Group B. Higher proportion (36%) of patients had the medical history of urticaria followed by atopic dermatitis (26%), asthma (23%) and rhinitis (23%). The commonest sensitizing food allergen was banana (68%) followed by sesame seeds (66%), lemon (45%), rice (31%), wheat (24%), cashew (23%) and peanut (21%). Among inhalants, house dust mite, *D. farinae* (83%) was the most prevalent sensitizing allergen followed by cockroach (79%), weed pollens (29-50%), tree pollen (16-29%), grass pollen mix (26%) and mold mix (25%). Less than 20% of patients tested positive to cow’s milk, tomato, spinach, aubergine, soybean, egg white, dog dander, legume mix, chicken, cat dander and fish. The geometric mean of total IgE was 943 kU/l (21->5000 kU/l, range 2-5000 kU/l).

## Conclusions

This is the first Indian sensitization data of this dignity analyzed by ImmunoCAP^®^ which provided useful native information of prevalent sensitizing Indian allergens that would improve cost effectiveness of allergy treatment and hence increase the quality of life of allergic patients in India. Phadiatop/fx5 revealed that the physicians’ diagnosis of IgE mediated allergy was accurate only in 59% of cases and thus highlights the importance of using allergy tests in conjunction with clinical findings for correct allergy diagnosis.

